# Median bias reduction in random-effects meta-analysis and meta-regression

**DOI:** 10.1177/0962280218771717

**Published:** 2018-05-02

**Authors:** Sophia Kyriakou, Ioannis Kosmidis, Nicola Sartori

**Affiliations:** 1 Department of Statistical Science, University College London, London, UK; 2 Department of Statistics, University of Warwick, Coventry, UK; 3 The Alan Turing Institute, London, UK; 4 Department of Statistical Sciences, University of Padova, Padova, Italy

**Keywords:** Adjusted score equations, heterogeneity, mean bias reduction, penalised likelihood, random effects

## Abstract

The reduction of the mean or median bias of the maximum likelihood estimator in regular parametric models can be achieved through the additive adjustment of the score equations. In this paper, we derive the adjusted score equations for median bias reduction in random-effects meta-analysis and meta-regression models and derive efficient estimation algorithms. The median bias-reducing adjusted score functions are found to be the derivatives of a penalised likelihood. The penalised likelihood is used to form a penalised likelihood ratio statistic which has known limiting distribution and can be used for carrying out hypothesis tests or for constructing confidence intervals for either the fixed-effect parameters or the variance component. Simulation studies and real data applications are used to assess the performance of estimation and inference based on the median bias-reducing penalised likelihood and compare it to recently proposed alternatives. The results provide evidence on the effectiveness of median bias reduction in improving estimation and likelihood-based inference.

## 1 Introduction

Meta-analysis is a core tool for synthesising the results from independent studies investigating a common effect of interest. One of the main challenges when combining results from multiple studies is the variability or heterogeneity in the design and the methods employed in each study. Accounting for and quantifying that heterogeneity is critical when drawing inferences about the common effect. In this direction, DerSimonian and Laird^1^ introduced the random-effects meta-analysis model, which expresses the heterogeneity between studies in terms of a variance component that can be estimated through standard estimation techniques.

Nevertheless, there is ample evidence that frequentist inference based on random-effects meta-analysis can be problematic in the usual meta-analytic scenario where the number of studies is small or moderate. Specifically, the estimation of the heterogeneity parameter can be highly imprecise, which in turn results in misleading conclusions.^2–4^ Examples of recently proposed methods that attempt to improve inference are the resampling^5^ and double resampling approaches,^6^ and the mean bias-reducing penalised likelihood (BRPL) approach in Kosmidis et al.^3^ Specifically, Kosmidis et al.^3^ show that maximisation of the BRPL results in an estimator of the heterogeneity parameter that has notably smaller bias than maximum likelihood (ML) with small loss in efficiency, and illustrate that BRPL-based inference outperforms its competitors in terms of inferential performance.

Kenne Pagui et al.^7^ show that under suitable conditions, third-order median unbiased estimators can be obtained by the solution of a suitably adjusted score equation. The components of such median bias-reduced estimators have, to third-order, the same probability of over- and under-estimating the true parameter. A key property of these estimators, not shared with the mean bias-reduced ones, is that any monotone component-wise transformation of the estimators results automatically in median bias-reduced estimators of the transformed parameters.^7^ Such equivariance property can be useful in the context of random-effects meta-analysis where the Fisher information and, hence, the asymptotic variances of various likelihood-based estimators depend only on the heterogeneity parameter.

In this paper, we derive the median bias-reducing adjusted score functions for random-effects meta-analysis and meta-regression. The adjusted score functions are found to correspond to a median BRPL, whose logarithm differs from the logarithm of the mean BRPL in Kosmidis et al.^3^ by a simple additive term that depends on the heterogeneity parameter. Since the adjustments to the score function for mean and median bias reduction are both of order *O*(1), the same arguments as in Kosmidis et al.^3^ are used to obtain a median BRPL ratio statistic with known asymptotic null distribution that can be used for carrying out hypothesis tests and constructing confidence regions or intervals for either the fixed-effect or the heterogeneity parameter. Simulation studies and real data applications are used to assess the performance of estimation and inference based on the median BRPL, and compare it to recently proposed alternatives, including the mean BRPL. The results provide evidence on the effectiveness of median bias reduction in improving estimation and likelihood-based inference.

The rest of the paper is organised as follows. Section 2 considers the cocoa intake dataset^8^ and a random-effects meta-analysis model as a motivational example that demonstrates how conclusions in frequentist inference may vary using different methods when the number of studies is small. Section 3 defines the random-effects meta-analysis and meta-regression model and establishes notation. Section 4 gives a formal statement of the proposed median bias-reducing adjusted score function and penalised likelihood for these models, gives the algorithm used for computing the median BRPL estimates, and briefly discusses the median BRPL ratio statistic used for inference. Section 5 revisits the example in Section 2 and gives some simulations comparing the median BRPL method to alternative approaches. Section 6 gives more extensive simulations under the random-effects meta-analysis model that evaluate the performance of median BRPL and compare it with that of ML and mean BRPL methods. An application to meat consumption data^9^ and a random-effects meta-regression model is given in Section 7. Section 8 concludes with a brief discussion, and Appendix 1 contains some technical details on the derivation of the median bias-reducing adjusted score functions.

## 2 Cocoa intake and blood pressure reduction data

**Figure 1.**
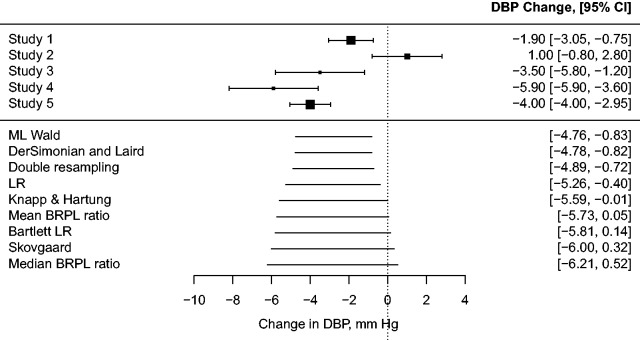
Forest plot of cocoa data.^8,10^ The outcomes from the five studies are reported in terms of the diastolic blood pressure (DBP) difference after two weeks of cocoa consumption. A negative change in DBP indicates favorable hypotensive cocoa actions. Squares represent the mean effect estimate for each study; the size of the square reflects the weight that the corresponding study exerts in the meta-analysis calculated as the within-study’s inverse variance. Horizontal line segments represent 95% Wald-type confidence intervals (CI) of the effect estimate of individual studies. In the bottom panel of the plot, horizontal line segments represent the corresponding 95% confidence interval as computed based on the Wald statistic using the ML estimates (ML Wald), the DerSimonian and Laird approach,^1^ double resampling,^6^ the LR statistic, the Knapp and Hartung^11^ method, the mean BRPL ratio statistic,^3^ the Bartlett-corrected LR statistic (Bartlett LR),^12^ the Skovgaard’s statistic, and the median BRPL ratio statistic. The confidence intervals *n* are ordered according to their length. The estimate of *β* has not been reported, as is commonly done in forest plots, because some of the methods considered (e.g. Skovgaard, Bartlett-corrected LR, and double resampling) are designed to produce directly *p*-values and/or confidence intervals and do not directly correspond to an estimation method.

Consider the setting in Bellio and Guolo^10^ who carry out a meta-analysis of five randomised controlled trials from Taubert et al.^8^ on the efficacy of two weeks of cocoa consumption on lowering diastolic blood pressure. Figure 1 is a forest plot with the estimated mean difference in diastolic blood pressure before and after cocoa intake from each study, and the associated 95% Wald-type confidence intervals. Four out of the five studies reported a reduction of diastolic blood pressure from cocoa intake.

The random-effects meta-analysis model is used to synthesise the evidence from the five studies. In particular, let *Y_i_* be the random variable representing the mean difference in the diastolic blood pressure after two weeks of cocoa intake in the *i*th study. We assume that $Y_{1},\ldots ,Y_{5}$ are independent random variables, where *Y_i_* has a Normal distribution with mean the overall effect *β* and variance σ^i2+ψ, with σ^i2 the estimated standard error of the effect from the *i*th study and *ψ* the heterogeneity parameter.

The forest plot in Figure 1 has been enriched with several nominally 95% confidence intervals for *β* using various alternative methods. As is apparent, the conclusions when testing the hypothesis *β* = 0 can vary depending on the method used. More specifically, the Wald test using the ML estimates, the DerSimonian and Laird method,^1^ double resampling,^6^ and the likelihood ratio (LR) test give evidence that there is a relationship between cocoa consumption and diastolic blood pressure, with *p*-values 0.005, 0.006, 0.016, 0.030, respectively. On the other hand, Knapp and Hartung’s method,^11^ the mean BRPL ratio,^3^ the Bartlett-corrected LR,^12^ and Skovgaard’s test suggest that the evidence that cocoa consumption affects diastolic blood pressure is weaker, with *p*-values of 0.050, 0.053, 0.058, and 0.067, respectively.

## 3 Random-effects meta-regression model

Let *y_i_* and σ^i2 denote the estimate of the effect from the *i*th study (i=1,…,K) and the associated within-study variance, respectively, and $x_{i}=(x_{i1},\ldots ,x_{\mathrm{ip}}{)}^{\text{T}}$ denote a *p*-vector of study-specific covariates that can be used to account for the heterogeneity across studies.

The within-study variances σ^i2 are usually assumed to be estimated well enough to be considered as known and equal to the values reported in each study. Then the observations $y_{1},\ldots ,y_{K}$ are assumed to be realisations of the random variables $Y_{1},\ldots ,Y_{K}$, which are independent conditionally on independent random effects $U_{1},\ldots ,U_{K}$. The conditional distribution of *Y_i_* given *U_i_* = *u_i_* is N(ui+xiTβ,σ^i2), where *β* is an unknown *p*-dimensional vector of fixed effects. The random effects $U_{1},\ldots ,U_{K}$ are typically assumed to be independent with *U_i_* having a N(0,ψ) distribution, where *ψ* is a parameter that attempts to capture the unexplained between-study heterogeneity. In matrix notation, the random-effects meta-regression model has

(1) Y=Xβ+U+ε

where $Y=(Y_{1},\ldots ,Y_{K}{)}^{\text{T}}$, *X* is the *K* × *p* model matrix with $x_{i}^{\text{T}}$ in its *i*th row, and $\varepsilon =(\varepsilon_{1},\ldots ,\varepsilon_{K}{)}^{\text{T}}$ is a vector of independent errors each with a N(0,σ^i2) distribution and independent of $U=(U_{1},\ldots ,U_{K}{)}^{\text{T}}$. Under this specification, the marginal distribution of *Y* is multivariate normal with mean Xβ and variance–covariance matrix Σ^+ψIK, where *I_K_* is the K×K identity matrix and Σ^=diag(σ^12,…,σ^K2). The random-effects meta-analysis results as a special case of meta-regression, by setting *X* to be a column of ones.

The log-likelihood function for $\theta =(\beta^{\text{T}},\psi {)}^{\text{T}}$ is $l(\theta )=\{\text{log}|W(\psi )|-R(\beta {)}^{\text{T}}W(\psi )R(\beta )\}/2$, where |W(ψ)| denotes the determinant of W(ψ)=(Σ^+ψIK)-1 and R(β)=y-Xβ. The gradient of the log-likelihood (score function) is

(2) $$s(\theta )=\begin{array}{c} (\begin{array}{c} X^{\text{T}}W(\psi )R(\beta )\\ \frac{1}{2}\{R(\beta {)}^{\text{T}}W(\psi {)}^{2}R(\beta )-\text{tr}[W(\psi )]\} \end{array} \end{array})$$

and the ML estimator θ^=(β^T,ψ^)T is obtained as the solution of $s(\theta )=0_{p+1}$, where $0_{p}$ denotes a *p*-dimensional vector of zeros.

## 4 Median bias reduction

### 4.1 The method

A popular method for reducing the mean bias of ML estimates in regular statistical models is through the adjustment of the score equation.^13,14^ Kenne Pagui et al.^7^ propose an extension of the adjusted score equation approach which can be used to obtain median bias-reduced estimators. Specifically, under the model, the new estimator has a distribution with median closer to the “true” parameter value than the ML estimator. Kenne Pagui et al.^7^ consider the median as a centering index for the score, and the adjusted score function for median bias reduction then results by subtracting from the score its approximate median, obtained using a Cornish-Fisher asymptotic expansion.

Let $j(\theta )=-\partial^{2}l(\theta )/\partial \theta \partial \theta^{\text{T}}$ be the observed information matrix (see Appendix 1 for its expression), and i(θ) be the expected information matrix

(3) $$i(\theta )=E_{\theta }(j(\theta ))=\begin{array}{c} (\begin{array}{cc} X^{\text{T}}W(\psi )X & 0_{p}\\ 0_{p}^{\text{T}} & \frac{1}{2}\text{tr}[W(\psi {)}^{2}] \end{array} \end{array})$$

with *t*th column $i_{t}(\theta )$. Let also $i^{t}(\theta )$ and $i^{\mathrm{tt}}(\theta )$ be the *t*th column and the *t*th diagonal element of $\{i(\theta ){\}}^{-1}$, with t∈{1,…,p+1}. Kenne Pagui et al.^7^ show that a median bias-reduced estimator θ^† can be obtained by solving an adjusted score equation of the form $s^{†}(\theta )=s(\theta )+A^{†}(\theta )=0$, where the additive adjustment to $A^{†}(\theta )$ is *O*(1), in the sense that $A^{†}(\theta )$ is bounded in absolute value by a fixed constant after a sufficiently large value of *K*. The median bias-reducing adjustment $A^{†}(\theta )$ has *t*th element

(4) $$A_{t}^{†}(\theta )=\frac{1}{2}\text{tr}[\{i(\theta ){\}}^{-1}(P_{t}(\theta )+Q_{t}(\theta ))]-\{i_{t}(\theta ){\}}^{\text{T}}K^{†}(\theta ).$$

The quantities $P_{t}(\theta )=E_{\theta }[s(\theta )s^{\text{T}}(\theta )s_{t}(\theta )]$ and $Q_{t}(\theta )=E_{\theta }[-j(\theta )s_{t}(\theta )]$ in equation (4) are those introduced by Kosmidis and Firth^14^ for mean bias-reduction, and $K^{†}(\theta )$ is a (p+1)-vector with *t*th element $K_{t}^{†}(\theta )=\{i^{t}(\theta ){\}}^{\text{T}}K_{t}(\theta )$, where $K_{t}(\theta )$ is another (p+1)-vector with *u*th element

$$K_{\mathrm{tu}}(\theta )=\text{tr}[\frac{i^{t}(\theta )\{i^{t}(\theta ){\}}^{\text{T}}}{i^{\mathrm{tt}}(\theta )}(\frac{1}{3}P_{u}(\theta )+\frac{1}{2}Q_{u}(\theta ))]$$

In the context of meta-regression values of *t* and *u* in {1,…,p} correspond to the elements of parameter *β*, and t,u=p+1 correspond to parameter *ψ*. Given that $A^{†}(\theta )$ is of order *O*(1), θ^† has the same asymptotic distribution as θ^,^7^ i.e. multivariate normal with mean *θ* and variance-covariance matrix $\{i(\theta ){\}}^{-1}$, which can be consistently estimated with {i(θ^†)}-1.

After some algebra (see Appendix 1 for details), the median bias-reducing adjustment for the random-effects meta-analysis and meta-regression models has the form

(5) $$A^{†}(\theta )=\begin{array}{c} (\begin{array}{c} 0_{p}\\ \frac{1}{2}\text{tr}[W(\psi )H(\psi )]+\frac{1}{3}\frac{\text{tr}[W(\psi {)}^{3}]}{\text{tr}[W(\psi {)}^{2}]} \end{array}) \end{array}$$

where $H(\psi )=X(X^{\text{T}}W(\psi )X{)}^{-1}X^{\text{T}}W(\psi )$. Substituting equation (5) in the expression for $s^{†}(\theta )$ gives that the median bias-reducing adjusted score functions for *β* and *ψ* are $s_{\beta }^{†}(\theta )=s_{\beta }(\theta )$ and

$$s_{\psi }^{†}(\theta )=s_{\psi }(\theta )+\frac{1}{2}\text{tr}[W(\psi )H(\psi )]+\frac{1}{3}\frac{\text{tr}[W(\psi {)}^{3}]}{\text{tr}[W(\psi {)}^{2}]}$$

respectively.

### 4.2 Computation of median bias-reduced estimator

A direct approach for computing the estimator θ^†=(β^†T,ψ^†)T is through a modification of the two-step iterative process in Kosmidis et al.^3^ At the *j*th iteration (j=1,2,…)

1. Calculate $\beta^{\left(j\right)}$ by weighted least squares as $\beta^{\left(j\right)}=(X^{\text{T}}W(\psi^{\left(j-1\right)})X{)}^{-1}X^{\text{T}}W(\psi^{\left(j-1\right)})y$
2. Solve $s_{\psi }^{†}(\theta^{\left(j\right)}(\psi ))=0$ with respect to *ψ*, where $\theta^{\left(j\right)}(\psi )=(\beta^{(j)\text{T}},\psi {)}^{\text{T}}$.

In the above steps, $\beta^{\left(j\right)}$ is the candidate value for β^† at the *j*th iteration and $\psi^{\left(j-1\right)}$ is the candidate value for ψ^† at the (j-1)th iteration. The equation in step 2 is solved numerically, by searching for the root of the function $s_{\psi }^{†}(\beta^{\left(j\right)},\psi )$ in a predefined positive interval. For the computations in this manuscript, we use the DerSimonian and Laird^1^ estimate of *ψ* as starting value $\psi^{\left(0\right)}$. The iterative process is then repeated until the components of the score function $s^{†}(\theta )$ are all less than $\varepsilon =1\times 10^{-6}$ in absolute value at the current estimates.

### 4.3 Median bias-reducing penalised likelihood

Although it is not generally true that $s^{†}(\theta )$ is the gradient of a suitable penalised log-likelihood, in this case $s^{†}(\theta )$ is the gradient of the median BRPL

(6) $$l^{†}(\theta )=l(\theta )-\frac{1}{2}\text{log}|X^{\text{T}}W(\psi )X|-\frac{1}{6}\text{log}[\text{tr}(W(\psi {)}^{2})]$$

Hence, θ^† is also the maximum median BRPL estimator. The median BRPL in equation (6) differs from the mean BRPL derived in Kosmidis et al.^3^ by the term $-\text{log}[\text{tr}(W(\psi {)}^{2})]/6$.

An advantage of the median BRPL estimators over mean BRPL ones is that the former are equivariant under monotone component-wise parameter transformations.^7^ In the context of random-effects meta-analysis and meta-regression, this equivariance implies that not only we get a median bias-reduced estimator of *ψ*, but we also get median bias-reduced estimates of the standard errors for *β* by calculating the square roots of the diagonal elements of $\{i(\theta ){\}}^{-1}$ in equation (3) at $\psi^{†}$. This is because i(θ) is a function of *ψ* only, and moreover the square roots of the diagonal elements of $\{i(\theta ){\}}^{-1}$ are monotone functions of *ψ*.

### 4.4 Penalised likelihood-based inference

For inference about either the components of the fixed-effect parameters *β* or the between-study heterogeneity *ψ*, we propose the use of the median BRPL ratio. If $\theta =(\tau^{\text{T}},\lambda^{\text{T}}{)}^{\text{T}}$ and λ^τ† is the maximiser of $l^{†}(\theta )$ for fixed *τ*, then the same arguments as in Kosmidis et al.^3^ can be used to show that the logarithm of the median BRPL ratio statistic

(7) 2{l†(τ^†,λ^†)-l†(τ,λ^τ†)}

has a $\chi_{\dim(\tau )}^{2}$ asymptotic distribution, as *K* goes to infinity. Specifically, the adjustment to the score function is additive and of order *O*(1). As a result, the extra terms in the asymptotic expansion of the logarithm of the median BRPL that depend on the penalty and its derivatives disappear as information increases, and the expansion has the same leading term as that of the log-likelihood (see, for example, Pace and Salvan^15^ Section 9.4).

## 5 Cocoa intake and blood pressure reduction data (revisited)

The ML estimate, the maximum mean BRPL estimate and the maximum median BRPL estimate of the heterogeneity parameter in the meta-analysis model in Section 2 are ψ^=4.199, ψ^*=5.546, and ψ^†=6.897, respectively. The estimates of the common effect are β^=-2.799,β^*=-2.811, and β^†=-2.818, with standard errors 1.000, 1.128, and 1.242, respectively. The bias-reduced estimates of *ψ* and, as a consequence, the corresponding estimated standard errors for *β* are larger than their ML counterparts, which is typical in random-effects meta-analysis. The iterative process used for computing the ML, maximum mean BRPL, and maximum median BRPL estimates converged in 4, 5, and 11 iterations, respectively. The computational run-time for the two-step iterative process which computes the ML, maximum mean BRPL, and maximum median BRPL estimates is $1.1\times 10^{-2},1.8\times 10^{-2}$, and $1.1\times 10^{-2}$ seconds, respectively.

**Figure 2.**
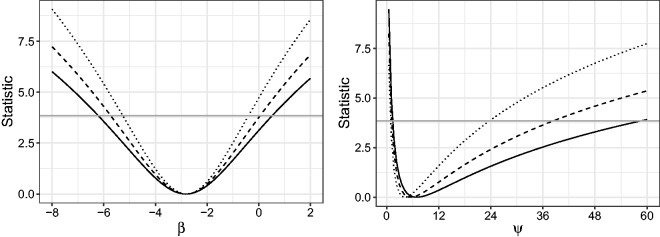
Plot of LR (dotted), mean BRPL (dashed) and median BRPL (solid) ratio statistic in equation (7) when *τ* is *β* (left) and *ψ* (right). The horizontal line is the 95% quantile of the limiting $\chi_{1}^{2}$ distribution, and its intersection with the values of the statistics results in the endpoints of the corresponding 95% confidence intervals.

Figure 2 shows the value of LR, mean BRPL and median BRPL ratio statistic in equation (7) for a range of values of *τ*, when *τ* is either *β* or *ψ*. Here and in the following simulation studies, we compare median BRPL ratio statistic with only LR and mean BRPL ratio statistics because the mean BRPL ratio statistic is a strong competitor against other alternatives in terms of inferential performance.^3^ The horizontal line in Figure 2 is the 95% quantile of the limiting $\chi_{1}^{2}$ distribution, and its intersection with the values of the statistics results in the endpoints of the corresponding 95% confidence intervals. For both *β* and *ψ*, the confidence intervals based on the LR statistic are the narrowest and the confidence intervals based on the median BRPL ratio statistic are the widest. Specifically, the 95% confidence intervals for *β* are (-6.21,0.52),(-5.73,0.05), and (-5.26,-0.40) for the median BRPL ratio statistic, mean BRPL ratio statistic, and LR statistic, respectively. The corresponding 95% confidence intervals for *ψ* are (1.4,58.0),(1.0,38.5), and (1.1,23.5), respectively. Contrary to the LR test, the mean BRPL and median BRPL ratio tests suggest that there is only weak evidence that cocoa consumption affects diastolic blood pressure with *p*-values of 0.053 and 0.077.

**Figure 3.**
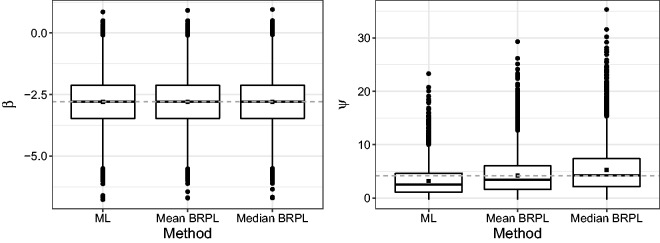
Boxplots for the ML, the maximum mean BRPL, and the maximum median BRPL estimates of *β* and *ψ* as calculated from 10,000 simulated samples under the ML fit using the cocoa data.^8,10^ The square point is the empirical mean of the estimates. The dashed grey horizontal line is at the parameter value used to generate the data.

In order to further investigate the performance of the three approaches to estimation and inference, we performed a simulation study where we simulated 10,000 independent samples from the random-effects meta-analysis model with parameter values set to the ML estimates reported earlier, i.e. $\beta_{0}=-2.799$ and $\psi_{0}=4.199$. Figure 3 shows boxplots of the estimates of *β* and *ψ* calculated from each of the 10,000 simulated samples. The distributions of the three alternative estimators for *β* are similar. On the other hand, the ML estimator of *ψ* has a large negative mean bias, maximum median BRPL tends to over-correct for that bias, while maximum mean BRPL almost fully corrects for the bias of ML estimator. The distribution of the median BRPL estimates has the heaviest right tail. The simulation-based estimates of the probabilities of underestimation for *ψ*, Pψ0(ψ^≤ψ0), Pψ0(ψ^*≤ψ0) and Pψ0(ψ^†≤ψ0) are 0.708, 0.591, and 0.493 for the ML, maximum mean BRPL, and maximum median BRPL, respectively, illustrating how effective maximising the median BRPL in equation (6) is in reducing the median bias of the maximum likelihood estimator of *ψ*.

The simulated samples were also used to calculate the empirical *p*-value distribution for the two-sided tests that each parameter is equal to the true values based on the LR statistic, the mean BRPL ratio statistic, and the median BRPL ratio statistic. Table 1 shows that the empirical *p*-value distribution for the mean and median BRPL ratio statistics are closest to uniformity, with the latter being slightly more conservative than the former. The coverage probability of the 95% confidence intervals of *β* based on the mean BRPL ratio and the median BRPL ratio are notably closer to the nominal level than those based on the likelihood ratio. Specifically, the coverage probabilities for *β* are 88%, 93%, and 96% for LR, mean BRPL ratio, and median BRPL ratio, respectively, and the corresponding coverage probabilities for *ψ* are 88%, 94%, and 96%, respectively.

**Table 1.** Empirical *p*-value distribution (%) for the tests based on the LR statistic, the mean BRPL ratio statistic, and the median BRPL ratio statistic in the cocoa data^8,10^ setting.

α×100	1.0	2.5	5.0	10.0	25.0	50.0	75.0	90.0	95.0	97.5	99.0
LR	5.7	8.4	11.8	18.2	34.6	57.8	79.2	91.8	96.1	98.0	99.3
Mean BRPL ratio	1.6	3.7	6.7	12.2	28.4	52.8	76.6	90.9	95.5	97.9	99.1
Median BRPL ratio	0.6	1.8	4.1	8.6	23.1	48.5	74.2	90.0	95.0	97.5	99.1

## 6 Simulation study

More extensive simulations under the random-effects meta-analysis model (1) are performed here using the design in Brockwell and Gordon.^16^ Specifically, the data $y_{i},i\in \{1,\ldots ,K\}$ are simulated from model (1) with true fixed-effect parameter β=0.5. The within-study variances σ^i2 are independently generated from a $\chi_{1}^{2}$ distribution and are multiplied by 0.25 before restricted to the interval (0.009,0.6). Eleven values of the between-study variance *ψ* ranging from 0 to 0.1 are chosen, and the number of studies *K* ranges from 5 to 200. For each combination of *ψ* and *K* considered, we simulated 10,000 data sets initialising the random number generator at a common state. The within-study variances were generated only once and kept fixed while generating the samples.

Zeng and Lin^6^ compared the performance of their proposed double resampling method with the DerSimonian and Laird^1^ method, the profile likelihood method in Hardy and Thompson,^17^ and the resampling method in Jackson and Bowden^5^ and showed that the double resampling method improves the accuracy of statistical inference. Based on these results, Kosmidis et al.^3^ compared the performance of their mean BRPL approach with the double resampling method and illustrated that the former results in confidence intervals with coverage probabilities closer to the nominal level than the alternative methods.

We take advantage of the results reported in Zeng and Lin^6^ and Kosmidis et al.^3^ and evaluate the performance of estimation and inference based only on the median BRPL with that based on the likelihood and the mean BRPL. The estimators of the fixed and random-effect parameters obtained from the three methods are calculated using variants of the two-step algorithm described in Section 4.2. In the second step of the algorithm, the candidate values for the ML, and maximum mean and median BRPL estimators of the between-study variance *ψ* are calculated by searching for the root of the partial derivatives of l(θ), $l^{*}(\theta )$, and $l^{†}(\theta )$ with respect to *ψ*, in the interval (0, 3).

**Figure 4.**
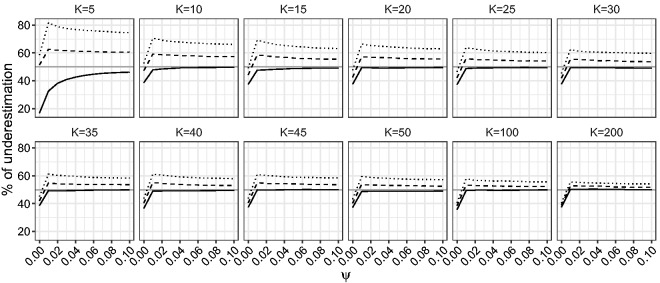
Empirical percentage of underestimation for *ψ* for random-effects meta-analysis. The percentage of underestimation is calculated for K∈{5,10,15,20,25,30,35,40,45,50,100,200} and for increasing values of *ψ* in the interval [0,0.1]. The curves correspond to the maximum median BRPL (solid), maximum mean BRPL (dashed), and ML (dotted) estimators. The grey horizontal line is at the target of 50% underestimation.

**Figure 5.**
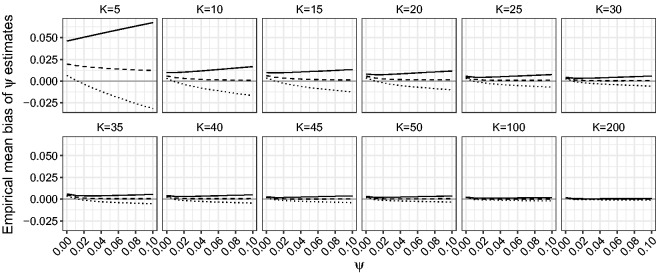
Empirical mean bias of *ψ* estimates for random-effects meta-analysis. The mean bias is calculated for K∈{5,10,15,20,25,30,35,40,45,50,100,200} and for increasing values of *ψ* in the interval [0,0.1]. The curves correspond to the maximum median BRPL (solid), maximum mean BRPL (dashed), and ML (dotted) estimators. The grey horizontal line is at zero.

First, we compare the performance of the ML, maximum mean BRPL and maximum median BRPL estimators in terms of percentage of underestimation. Figure 4 shows that the median bias-reducing adjustment is the most effective in reducing median bias even for small values of *K*. As expected, the ML and maximum mean BRPL estimators also approach the limit of 50% underestimation as *K* grows, with the latter being closer to 50% than the former. Figure 5 shows that maximum median BRPL is also effective in reducing the mean bias of the ML estimator of *ψ* but only for moderate to large values of *K*, while maximum mean BRPL results in estimators with the smallest bias.

**Figure 6.**
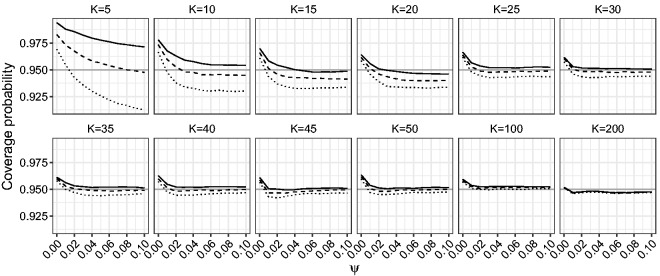
Empirical coverage probabilities of one-sided (right) confidence intervals for *β* for random-effects meta-analysis. The empirical coverage is calculated for increasing values of *ψ* in the interval [0,0.1] and for K∈{5,10,15,20,25,30,35,40,45,50,100,200}. The curves correspond to nominally 95% internals based on the median BRPL ratio (solid), the mean BRPL ratio (dashed), and the LR (dotted). The grey horizontal line is at the 95% nominal level.

**Figure 7.**
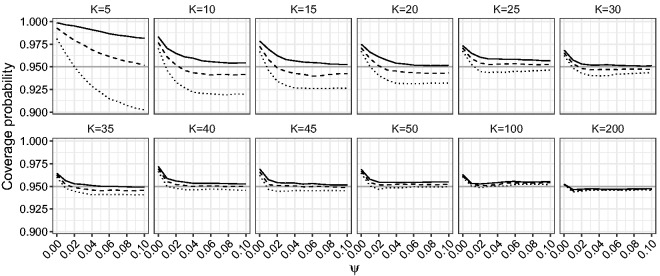
Empirical coverage probabilities of two-sided confidence intervals for *β* for random-effects meta-analysis. The empirical coverage is calculated for increasing values of *ψ* in the interval [0,0.1] and for K∈{5,10,15,20,25,30,35,40,45,50,100,200}. The curves correspond to nominally 95% confidence intervals based on the median BRPL ratio (solid), the mean BRPL ratio (dashed), and the LR (dotted). The grey horizontal line is at the 95% nominal level.

**Figure 8.**
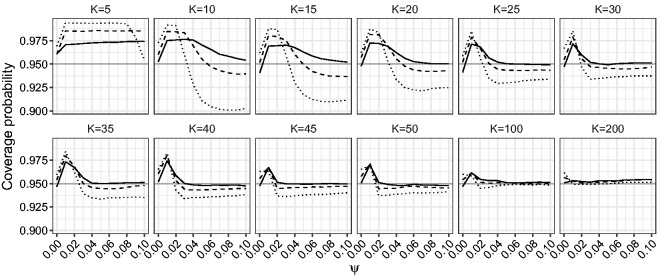
Empirical coverage probabilities of two-sided confidence intervals for *ψ* for random-effects meta-analysis. The empirical coverage is calculated with β=0.5 and for increasing values of *ψ* in the interval [0,0.1] and for K∈{5,10,15,20,25,30,35,40,45,50,100,200}. The curves correspond to nominally 95% confidence intervals based on the median BRPL ratio (solid), the mean BRPL ratio (dashed), and the LR (dotted). The grey horizontal line is at the 95% nominal level.

Figures 6 and 7 show the estimated coverage probability for the one-sided and two-sided confidence intervals for *β* based on the LR, mean BRPL ratio and median BRPL ratio statistics at the 95% nominal level. Figure 8 shows the estimated coverage probability for the two-sided confidence intervals for *ψ* based on the LR, mean BRPL ratio and median BRPL ratio statistics at the 95% nominal level. For small values of *ψ* or small and moderate number of studies *K*, the empirical coverage of the intervals is larger than the nominal 95% level. In general, the confidence intervals based on mean and median BRPL ratio have empirical coverage that is closer to the nominal level with the latter having generally better coverage. The differences between the three methods diminish as the number of studies *K* increases.

**Figure 9.**
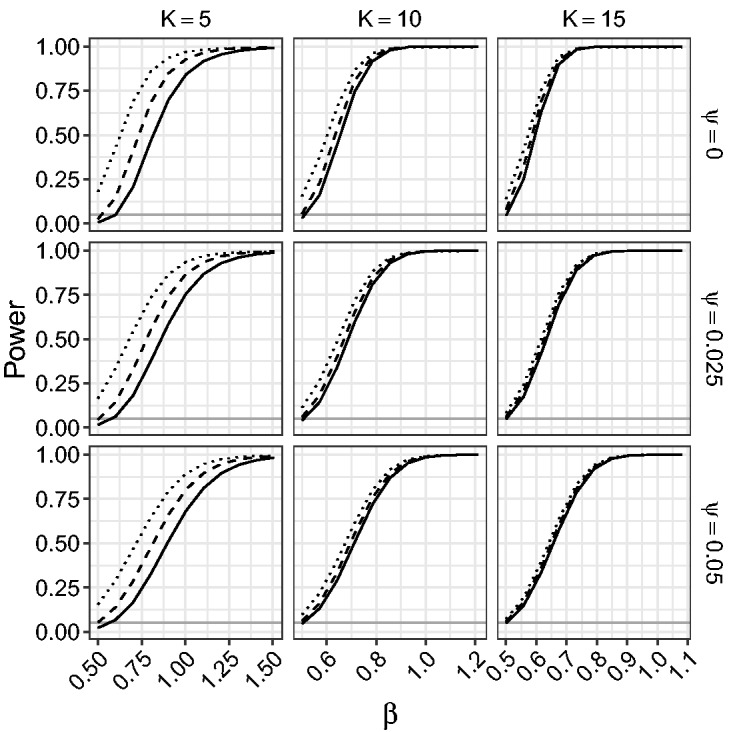
Empirical power of the likelihood-based tests of asymptotic level 0.05 for random-effects meta-analysis for testing β=0.5. The empirical power is calculated for increasing values of *β*, for K∈{5,10,15} and ψ∈{0,0.025,0.05}. The curves correspond to median BRPL ratio (solid), mean BRPL ratio (dashed), and LR (dotted) tests. The grey horizontal line is at the 5% nominal size.

**Figure 10.**
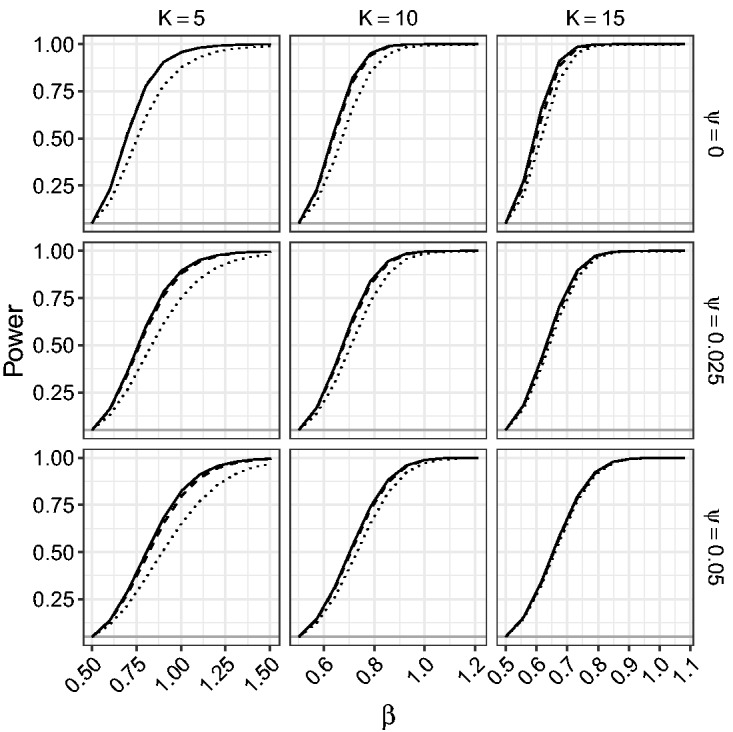
Empirical power of the likelihood-based tests of exact level 0.05 for random-effects meta-analysis for testing β=0.5. The empirical power is calculated for increasing values of *β*, for K∈{5,10,15} and ψ∈{0,0.025,0.05}. The curves correspond to median BRPL ratio (solid), mean BRPL ratio (dashed), and LR (dotted) tests. The grey horizontal line is at the 5% nominal size.

Figures 9 and 10 give the power of the LR, the mean BRPL ratio, and the median BRPL ratio tests for testing the null hypothesis β=0.5 against various alternatives. Specifically, we simulated 10,000 data sets under the alternative hypothesis that parameter *β* is equal to $b=0.5+\delta K^{-1/2}$, where *δ* ranges from 0 to 2.25. In Figure 9 the power is calculated using critical values of the asymptotic null $\chi_{1}^{2}$ distribution of the statistics. In Figure 10, the power is calculated using critical values based on the exact null distribution of each statistic, obtained by simulation under the null hypothesis. In this way, the three tests are calibrated to have size 5%.

Figure 9 shows that the three tests have monotone power and for small values of *K* the LR test yields the largest power. This is because the LR test is oversized, while the mean and median BRPL ratio tests are slightly more conservative and this conservativeness comes at the cost of lower power. As the number of studies *K* increases, the three tests approach the nominal size and provide similar power. The use of the exact critical values in Figure 10 allows us to compare the performance of the tests without letting the oversizing or the conservativeness of a test skew the power results. Figure 10 shows that the power of the median BRPL ratio test is almost identical to that of the mean BRPL ratio test, and both tests have larger power than the LR test. Again, inference based on either of the two penalised likelihoods becomes indistinguishable from classical likelihood inference as the number of studies increases.

Across all *ψ* and *K* values considered, the average number of iterations taken per fit for the two-step iterative process to converge is 6.20, 5.75, and 5.86 iterations for ML, maximum mean BRPL, and maximum median BRPL, respectively. The average computational run-times for ML, maximum mean BRPL, and maximum median BRPL are 0.005 s, 0.021 s, and 0.017 s, respectively. Figures 1 and 2 in the Supplementary material show the average number of iterations and the average computational run-time taken per fit for the two-step iterative process to converge for each value of *K* and *ψ* used in the simulation study. The results show that in all cases estimation is achieved rapidly and after a small number of iterations for all three methods, with only negligible overhead with the two bias reducing methods.

## 7 Meat consumption data

**Figure 11.**
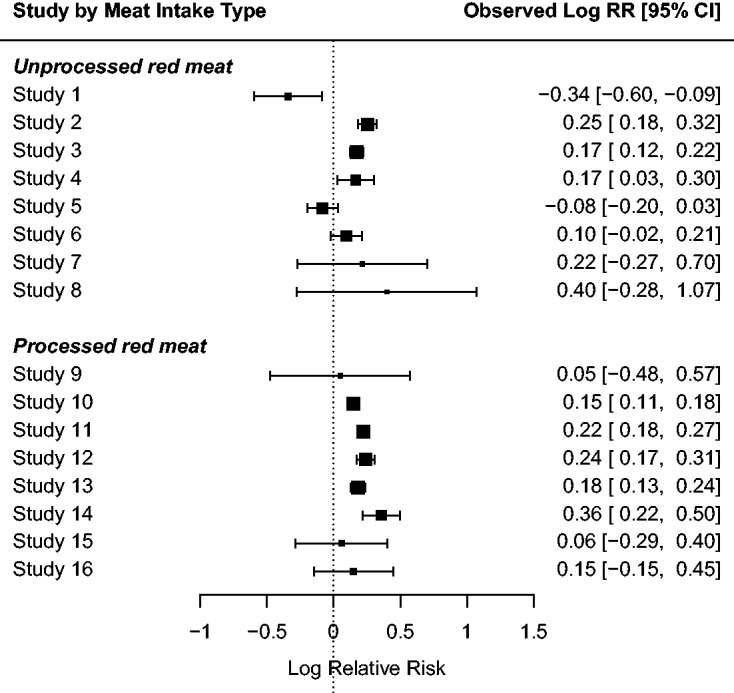
The meat consumption data.^9^ Outcomes from 16 studies are reported in terms of the logarithm of the relative risk (Log RR) of all-cause mortality for the highest versus lowest category of unprocessed red meat, and processed meat consumption. Squares represent the mean effect estimate for each study; the size of the square reflects the weight that the corresponding study exerts in the meta-analysis. Horizontal lines represent 95% Wald-type confidence intervals (CI) of the effect estimate of individual studies.

Larsson and Orsini^9^ investigate the association between meat consumption and relative risk of all-cause mortality. The data consists of 16 prospective studies, eight of which are about unprocessed red meat consumption and eight about processed meat consumption. Figure 11 displays the information provided by each study in the meta-analysis. The results from the studies point towards the conclusion that high consumption of red meat, in particular processed red meat, is associated with higher all-cause mortality.

We consider the random-effects meta-regression model, assuming that *Y_i_* has a N(β0+β1xi,σ^i2+ψ), where *Y_i_* is the random variable representing the logarithm of the relative risk reported in the *i*th study, and *x_i_* takes value 1 if the consumption in the *i*th study is about processed red meat and 0 if it is about unprocessed meat (i=1,…,16). Table 2 gives the ML estimates, the mean BRPL estimates, and the median BRPL estimates of *β*_0_, *β*_1_ and *ψ*, along with the corresponding standard errors for *β*_0_ and *β*_1_. The median BRPL estimate of *ψ* and the standard errors of the fixed-effect parameters are the largest. The iterative process for computing the ML, maximum mean BRPL, and maximum median BRPL estimates converged in eight, nine, and twelve iterations, in $1.2\times 10^{-2},2.4\times 10^{-2}$, and $1.5\times 10^{-2}$ seconds, respectively.

**Table 2.** ML, maximum mean BRPL, and maximum median BRPL estimates of the model parameters for the meat consumption data.^9^

Method	*β*_0_	*β*_1_	*ψ*
ML	0.099 (0.044)	0.106 (0.061)	0.009
	[−0.004,0.189]	[−0.022,0.244]	[0.003,0.030]
Maximum mean BRPL	0.095 (0.050)	0.110 (0.069)	0.012
	[−0.020,0.199]	[−0.040,0.264]	[0.003,0.042]
Maximum median BRPL	0.093 (0.052)	0.111 (0.072)	0.013
	[−0.027,0.203]	[−0.048,0.271]	[0.004,0.048]

Note: Standard errors are reported in parentheses. The 95% confidence intervals based on the LR, mean BRPL ratio and median BRPL ratio are reported in squared brackets.

The LR test indicates some evidence for a higher risk associated to the consumption of red processed meat with a *p*-value of 0.047. On the other hand, the mean and median BRPL ratio tests suggest that there is weaker evidence for higher risk, with *p*-values of 0.066 and 0.074, respectively. Skovgaard’s test also gives weak evidence for higher risk with *p*-value 0.073.

**Figure 12.**
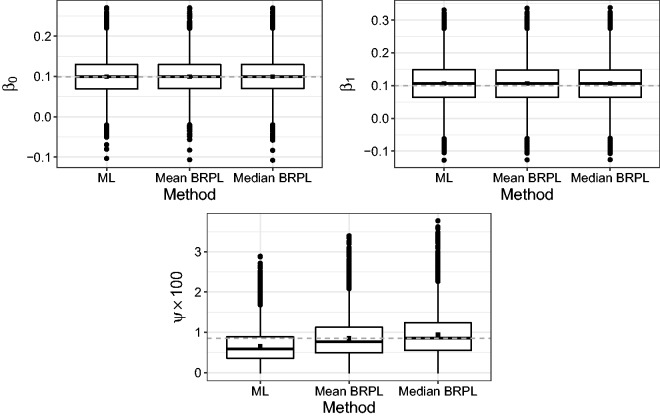
Boxplots for the ML, maximum mean BRPL, and maximum median BRPL estimates of *β*_0_, *β*_1_, and *ψ* as calculated from 10,000 simulated samples under the ML fit using the meat consumption data.^9^ The square point is the mean of the estimates obtained from each method. The dashed grey horizontal line is at the parameter value used to generate the data.

Similar to Section 5, we performed a simulation study in order to further investigate the performance of the three methods in a meta-regression context. We simulated 10,000 independent samples from the meta-regression model at the ML estimates reported in Table 2. Figure 12 shows boxplots of the estimates of *β*_0_, *β*_1_, and *ψ*. Maximum likelihood underestimates the parameter *ψ*, while mean BRPL and median BRPL almost fully compensate for the negative bias of ML estimates, with the latter having a slightly heavier right tail. The percentages of underestimation are 72.6%, 56.6%, and 49.9% for the ML, maximum mean BRPL, and maximum median BRPL estimators, respectively.

The simulated samples were also used to calculate the empirical *p*-value distribution for the tests based on the likelihood, mean BRPL and median BRPL ratio statistics. Table 3 shows that the empirical *p*-value distribution for the median BRPL ratio statistic is the one closest to uniformity.

**Table 3.** Empirical *p*-value distribution (%) for the tests based on the LR statistic, the mean BRPL ratio statistic, and the median BRPL ratio statistic using the meat consumption data.^9^

α×100	1.0	2.5	5.0	10.0	25.0	50.0	75.0	90.0	95.0	97.5	99.0
LR	2.2	4.5	7.7	13.1	28.0	50.0	71.7	86.6	92.1	95.3	97.7
Mean BRPL ratio	1.3	3.0	5.6	11.1	25.9	49.8	73.8	89.0	94.2	96.9	98.6
Median BRPL ratio	1.0	2.5	4.9	9.9	25.1	49.7	74.7	89.8	94.8	97.5	98.9

## 8 Concluding remarks

In this paper we derive the adjusted score equations for the median bias reduction of the ML estimator for random-effects meta-analysis and meta-regression models and describe the associated inferential procedures.

We show that the solution of the median bias-reducing adjusted score equations is equivalent to maximising a penalised log-likelihood. The logarithm of that penalised likelihood differs from the logarithm of the mean BRPL in Kosmidis et al.^3^ by a simple additive term. The computation of the maximum median BRPL estimators can be performed through a two-step iteration that involves a weighted least squares update and the solution of a nonlinear equation with respect to a scalar parameter, and which converges rapidly, as illustrated by the computational times and number of iterations reported in the paper. The reported times and number of iterations were computed using a workstation with 24 cores at 2.90 GHz and 80 GB memory running under the CentOS 7 operating system, using one core per data set.

Using various settings we were able to retrieve enough information on the performance of the maximum median BRPL estimators. All our simulation studies illustrate that use of the median BRPL succeeds in achieving median centering in estimation, and results in confidence intervals with good coverage properties. Furthermore, while tests based on the LR suffer from size distortions, the median BRPL ratio statistic results in tests with size and power properties, sometimes better to those of the mean BRPL ratio statistic in Kosmidis et al.^3^

The main advantage of the maximum median BRPL estimators from the maximum mean BRPL ones is their equivariance under monotone component-wise parameter transformations, which, in the case of random-effects meta-regression, leads to median bias-reduced standard errors.

As random-effect models are widely used in practice, the median BRPL method is likely to be useful in models with more complex random-effect structures, such as linear mixed models.

## Appendix 1

The observed information matrix for the random-effects meta-regression model (1) is

$$j(\theta )=\begin{array}{c} (\begin{array}{cc} X^{\text{T}}W(\psi )X & X^{\text{T}}W(\psi {)}^{2}R(\beta )\\ X^{\text{T}}W(\psi {)}^{2}R(\beta ) & R(\beta {)}^{\text{T}}W(\psi {)}^{3}R(\beta )-\frac{1}{2}\text{tr}[W(\psi {)}^{2}] \end{array}). \end{array}$$

For this model

$$P_{t}(\theta )=-Q_{t}(\theta )=\begin{array}{c} (\begin{array}{cc} 0_{p\times p} & X^{\text{T}}W(\psi {)}^{2}X_{t}\\ X^{\text{T}}W(\psi {)}^{2}X_{t} & 0 \end{array})(t=1,\ldots ,p), \end{array}$$

and

$$P_{p+1}(\theta )=\begin{array}{c} (\begin{array}{cc} X^{\text{T}}W(\psi {)}^{2}X & 0_{p}\\ 0_{p}^{\text{T}} & \text{tr}(W(\psi {)}^{3}) \end{array})\text{ and }Q_{p+1}(\theta )=(\begin{array}{cc} 0_{p\times p} & 0_{p}\\ 0_{p}^{\text{T}} & -\text{tr}(W(\psi {)}^{3}) \end{array}), \end{array}$$

where $0_{p\times p}$ is the *p* × *p* zero matrix and *X_t_* is the *t*th column of *X*. The median bias-reducing adjustment for *θ* is obtained by plugging the above expressions into equation (4). The sum $P_{t}(\theta )+Q_{t}(\theta )$ (t=1,…,p+1) is also given in the Appendix of Kosmidis et al.^3^
